# Selected Soybean Varieties Regulate Hepatic LDL-Cholesterol Homeostasis Depending on Their Glycinin:β-Conglycinin Ratio

**DOI:** 10.3390/antiox12010020

**Published:** 2022-12-22

**Authors:** Miguel Rebollo-Hernanz, Neal A. Bringe, Elvira Gonzalez de Mejia

**Affiliations:** 1 Department of Food Science and Human Nutrition, University of Illinois at Urbana-Champaign, Urbana, IL 61801, USA; 2 Benson Hill Company, St. Louis, MO 63132, USA

**Keywords:** β-conglycinin, cholesterol metabolism, glycinin, peptides, LDL oxidation, liver, protein, soybean

## Abstract

Clinical studies indicate that the consumption of soybean protein might reduce cholesterol and LDL levels preventing the development of atherosclerotic cardiovascular diseases. However, soybean variety can influence soybean protein profile and therefore affect soybean protein health-promoting properties. This study investigated the composition and effects of nineteen soybean varieties digested under simulated gastrointestinal conditions on hepatic cholesterol metabolism and LDL oxidation in vitro. Soybean varieties exhibited a differential protein hydrolysis during gastrointestinal digestion. Soybean varieties could be classified according to their composition (high/low glycinin:β-conglycinin ratio) and capacity to inhibit HMGCR (IC_50_ from 59 to 229 µg protein mL^−1^). According to multivariate analyses, five soybean varieties were selected. These soybean varieties produced different peptide profiles and differently reduced cholesterol concentration (43–55%) by inhibiting HMGCR in fatty-acid-stimulated HepG2 hepatocytes. Selected digested soybean varieties inhibited cholesterol esterification, triglyceride production, VLDL secretion, and LDL recycling by reducing ANGPTL3 and PCSK9 and synchronously increasing LDLR expression. In addition, selected soybean varieties hindered LDL oxidation, reducing the formation of lipid peroxidation early (conjugated dienes) and end products (malondialdehyde and 4-hydroxynonenal). The changes in HMGCR expression, cholesterol esterification, triglyceride accumulation, ANGPTL3 release, and malondialdehyde formation during LDL oxidation were significantly (*p* < 0.05) correlated with the glycinin:β-conglycinin ratio. Soybean varieties with lower glycinin:β-conglycinin exhibited a better potential in regulating cholesterol and LDL homeostasis in vitro. Consumption of soybean flour with a greater proportion of β-conglycinin may, consequently, improve the potential of the food ingredient to maintain healthy liver cholesterol homeostasis and cardiovascular function.

## 1. Introduction

Over the last few decades, the prevalence of overweight and obesity among adults has increased worldwide. Over 2 billion adults are overweight, while over 650 million are obese [1]. Metabolic syndrome is characterized by visceral obesity, hypertension, atherogenic dyslipidemia, insulin resistance, and glucose intolerance, raising the risk of metabolic-associated fatty liver disease (MAFLD) [2]. Convincing data suggest that systemic insulin resistance increases free fatty acid (FFA) transit from increased visceral adipose tissue into the liver and enhances hepatic de novo lipogenesis, resulting in fatty liver and enhanced hepatic insulin resistance [3]. The adipose tissue is essential for determining lipid fluxes to the liver in both fasting and fed conditions. When the demand for energy rises, FFAs are released by intracellular lipolysis of triglycerides from adipocyte lipid droplets [4]. A large proportion of FFAs is taken up by hepatocytes, which esterify them to form complex lipids. While FFAs are necessary metabolic substrates for cellular energy, an excess of FFAs can be harmful. Elevated FFA flux to the liver elicits increased hepatic cholesterol synthesis and lipid export in VLDL particles [5]. Effectively regulating key signaling pathways in the liver might prevent metabolic syndrome-associated diseases. Likewise, the plasma lipoprotein patterns in MAFLD are similar to those in metabolic syndrome and type 2 diabetes: elevated concentrations of low-density lipoproteins (LDL) and low concentrations of high-density lipoproteins (HDL). LDLs might be especially dangerous because they may easily enter the vascular intima, accelerating cholesterol accumulation in the atherosclerotic plaque [6]. Evidence suggests that free radical lipid peroxidation of polyunsaturated fatty acids found in phospholipids and cholesterol esters in lipoproteins plays a critical role in the development of atherosclerosis [7]. Angiopoietin-like 3 (ANGPTL3) has been described as a critical modulator of lipoprotein lipase (LPL) catalytic activity and lipid metabolism. Furthermore, ANGPTL3 regulates the hepatic lipid metabolism cell autonomously by increasing triglyceride-rich VLDL assembly and secretion and reducing hepatic LDL absorption via impaired LDL receptor (LDLR) expression [8]. ANGPTL3 is exclusively produced in the liver, its circulating levels are increased in MAFLD patients, and its inhibition might be essential in preventing atherosclerotic cardiovascular diseases [9,10].

Clinical evidence has proven the benefits of soybean protein in regulating lipid metabolism in the liver and other tissues. Clinical trials have demonstrated that soybean intake may offset several markers associated with MAFLD and lipid peroxidation [11]. Human studies have also indicated that soybean protein consumption reduces LDL levels while increasing HDL levels [12]. A recent meta-analysis has validated the effects of soybean protein intake on LDL cholesterol reduction [13]. Moreover, interventional studies have pointed out the protective effects of soybean protein consumption on preventing LDL oxidation [14]. In vivo experiments have pointed out that long-term soybean protein administration may attenuate steatosis in obese rats [15]. Based on cell culture experiments, soybean proteins and peptides exert interesting lipid-regulating and cholesterol-lowering activity in hepatocytes [16]. Soybean peptides, either from glycinin or β-conglycinin, regulate cholesterol synthesis by inhibiting 3-hydroxy-3-methylglutaryl coenzyme-A (HMG-CoA) reductase (HMGCR) and LDL liver uptake [17]. Soybean-prompted LDL absorption occurs through the increase in LDLR expression and proprotein convertase subtilisin/Kexin type 9 (PCSK9) inhibition [18,19]. Lunasin, a multifunctional soybean polypeptide, has also exhibited a cholesterol and LDL-lowering potential in vitro and in vivo [20]. However, non-significant lunasin-related effects have been proven in human clinical trials [21].

Varying protein profiles in different soybean varieties, primarily characterized by distinct glycinin:β-conglycinin ratios, have been associated with differential peptide release under simulated gastrointestinal conditions, different antioxidant properties in intestinal epithelial cells [22], and distinct adipogenic properties in mouse adipocytes [23,24]. Currently, there is a need to research the potential of various soybean protein profiles to regulate lipid metabolism and LDL-cholesterol homeostasis in the liver under MAFLD conditions owing to the limited knowledge on the topic. Therefore, our objective was to compare the composition and effects of nineteen soybean varieties digested under simulated gastrointestinal conditions in hepatic triglyceride and cholesterol metabolism and LDL oxidation and determine their effects on markers of cardiovascular health under conditions mimicking MAFLD. We hypothesized that soybean varieties with different glycinin:β-conglycinin ratios would regulate hepatocytes’ triglyceride and cholesterol homeostasis differently, regulating cholesterol concentration and secretion, and LDL oxidation and clearance in an in vitro cell model of MAFLD.

## 2. Materials and Methods

### 2.1. Materials

Nineteen different soybean varieties (V1: I245076, V2: I245077, V3: I245081, V4: I245083, V5: I245086, V6: I245087, V7: I245088, V8: I245089, V9: I245093, V10: I245094, V11: I245096, V12: I245097, V13: I245099, V14: I245103, V15: I245106, V16: I245107, V17: I245108, V18: GN#1, V19: GN#3) were supplied by Benson Hill (St. Louis, MO, USA) and stored at 4 °C until use. DC protein assay, 2× Laemmli buffer, 10× tris/glycine/SDS buffers, mini-PROTEAN^®^ TGX™ gels (4–20%, 15 well-comb, 15 μL), and Precision Plus Protein™ Dual Xtra standard were purchased from Bio-Rad (Hercules, CA, USA). Simply Blue Safe Stain was obtained from Invitrogen (Carlsbad, CA, USA). Bovine serum albumin (BSA), D-serine (98% purity), and simvastatin (97% purity) were used as standards and obtained from Sigma-Aldrich (St. Louis, MO, USA). Unless otherwise specified, all additional reagents were obtained from Sigma-Aldrich.

### 2.2. Defatted Soybean Flour

Soybean grains were processed in a coffee grinder. The milled soybean was sieved (1.16 mm) to achieve uniform particle size materials, and then preserved at −20 °C until required. The soybean flour was defatted using a Soxhlet extraction apparatus, as previously described [25].

### 2.3. Simulated Gastrointestinal Digestion

The simulated digestion was performed in accordance with the standardized INFOGEST protocol [26]. Defatted soybean flour (4 g) was combined with the oral mastermix in a 1:1 (*w/v*) ratio to generate an oral bolus. The oral bolus was then merged in a 1:1 (*w/v*) ratio with the gastric mastermix at pH 3, which contained simulated gastric fluid and pepsin (EC 3.4.23.1, 60 U mL^−1^), and digested for 2 h at 37 °C. The gastric phase was then mixed with the intestinal mastermix, including bile solution (10 mmol L^−1^), and pancreatin (100 U mL^−1^), and digested for 2 h at 37 °C. The duodenal phase was subsequently digested using a previously described method [27]. Pronase E (EC 3.4.24.4, from *Streptomyces griseus*, 4 mg mL^−1^, 3.5 U mg^−1^) was added, and the colonic phase was incubated at pH 8 and 37 °C for 1 h. Simultaneously, blank digestions were performed. The digestion was terminated by heating the digestion mixtures for 5 min at 100 °C. The bioaccessible fractions (soluble components) were retrieved by centrifugation at 4 °C, 3200× *g*, 40 min. The digestion supernatants were recovered, frozen at –80 °C, and freeze-dried.

### 2.4. Protein Quantification and Degree of Hydrolysis

Soluble protein was extracted from the non-digested defatted flours using the method previously described [25]. In brief, 75 mg of defatted flour was mixed with 1.5 mL of extraction solution (0.05 mol L^−1^ Tris-HCl buffer, pH 8.2). The suspension was homogenized and incubated in an ultrasonic bath at 40 °C for 70 min. The protein concentration of non-digested and digested soybean varieties flours was determined using the DC protein assay, as specified by the manufacturer (BioRad). The degree of hydrolysis (DH) was assessed as described [28]. In brief, the *o*-phthaldialdehyde (OPA) reagent was obtained by combining 10 mg of OPA 250 μL of ethanol, 9.8 mL of phosphate-buffered saline (PBS), and 20 μL of β-mercaptoethanol. Digested soybean (10 µL, 1 mg protein mL^−1^) was combined with 100 μL of OPA reagent and 140 μL of water. Serine was used as standard. The degree of hydrolysis was calculated using Equations (1) and (2).

(1) $$\mathrm{DH}\;\left(\%\right)=\frac{h}{h_{\mathrm{hot}}}\;\times \;100$$

(2) $$h=\frac{{\mathrm{Serine}-\mathrm{NH}}_{2}\;–\;\beta \;}{\alpha }$$

where h_hot_ = 7.8, Serine-NH_2_ was the serine concentration (mmol g protein^−1^) in digested soybean, β = 0.342, α = 0.970. 

### 2.5. Electrophoretic Profile by SDS-PAGE

**Figure A1:**
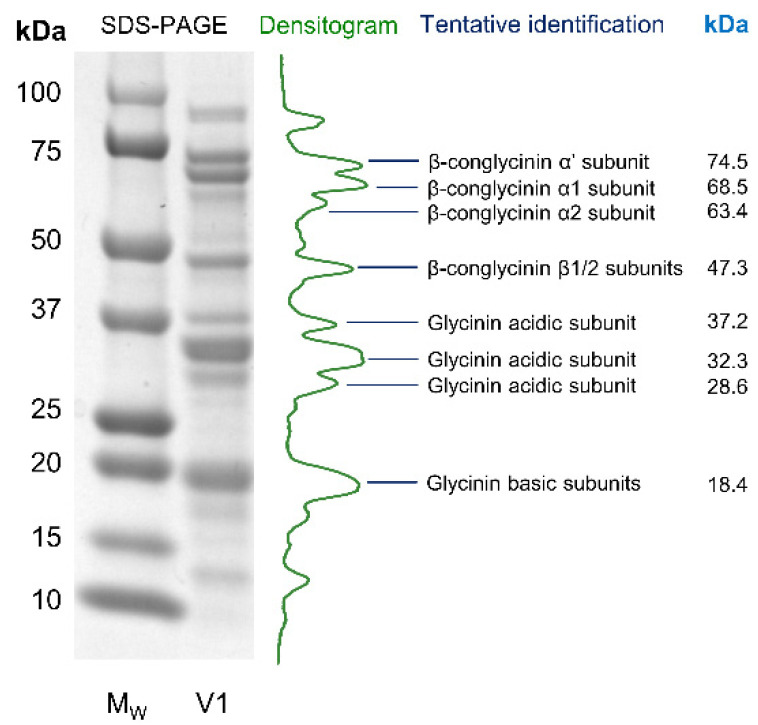
Representative SDS-PAGE electrophoresis and corresponding densitogram of soluble protein in soybean V1, and the tentative identification of main storage proteins (different of glycinin and β-conglycinin subunits).

The protein profile of non-digested and digested soybean flours was examined using SDS-PAGE. In brief, samples (20 μg of protein) were loaded in 4–20% Tris-Glycine gels. After that, electrophoresis was run for 35 min at 200 V and 400 mA. The gels were then rinsed three times with distilled water before being stained with SimplyBlue at room temperature for 1 h. The gels were subsequently destained with distilled water, and images were acquired using ImageQuant 800 Fluor colorimetric imaging. ImageJ was used to conduct densitometric measurements (National Institutes of Health, Bethesda, Maryland, USA). Proteins were tentatively identified by comparing their molecular weights to those of known proteins and their proportion in relation to the total protein concentration measured using the densitogram area under the curve (Appendix A, Figure A1). The glycinin:β-conglycinin ratio was calculated as the ratio between the sum of relative glycinin and β-conglycinin subunits proportion.

### 2.6. 3-Hydroxy-3-Methylglutaryl Coenzyme-A Reductase (HMGCR) Inhibition Assay

HMGCR was evaluated in plate, using an in vitro biochemical cell-free assay. The HMGCR (EC 1.1.1.34) assay kit was used according to the manufacturer’s instructions (Sigma-Aldrich, CS1090). In summary, NADPH (400 μmol L^−1^) and HMG-CoA substrate (0.3 mg mL^−1^) were combined with digested soybean varieties (10–3000 μg protein mL^−1^) or simvastatin (0.1–30 μg mL^−1^) in a UV compatible 96-well plate. To complete a final volume of 200 μL per well, PBS pH 7.4 was added. The analyses were initiated (time 0) by adding HMG-CoA reductase (2 μL of the enzyme stock solution; 0.50–0.70 mg protein mL^−1^) and incubated at 37 °C. The rates of NADPH consumed were monitored every 30 s for up to 20 min by reading the decrease in absorbance at 340 nm.

### 2.7. LC-ESI-MS/MS Peptide Sequencing, Bioinformatic Analysis, and Peptide Biological Activity

Peptides released from the selected soybean digested under gastrointestinal conditions were diluted in water (2 mg protein mL^−1^) and analyzed by LC-QTOF-MS/MS using Alliance 2795 HPLC system coupled to an Ultima mass spectrometer on the positive ion electrospray mode (+ESI) (Waters, Milford, MA, USA). The gradient mobile phase A contained 95% water, 5% acetonitrile, and 0.01% formic acid, while the gradient mobile phase B contained 95% acetonitrile, 5% water, and 0.1% formic acid. The flow rate was 400 µL min^−1^ and the PDA detector recorded the signal at 280 nm. All peaks with an intensity higher than 20% were examined using the MassLynx V4.1 program (Waters Corp, Milford, MA, USA). After obtaining the fragmentation pattern of each peak and determining the charge, MaxEnt3 was used to deconvolute representative mass spectra, which were then exported in the peptide sequencing tool to identify potential peptide sequences embedded within the soybean protein. Since distinguishing isoleucine (I) and leucine (L) is not possible, only isoforms with L were recovered, while peptide sequences having I instead of L are also possible. After that, I/L combinations were evaluated, and peptides were chosen based on the best parental protein prediction. The BLAST database (https://blast.ncbi.nlm.nih.gov/Blast.cgi, accessed on 16 December 2022) was used to compare the sequences obtained from digested soybean varieties to previously published protein sequences. Mass, isoelectric point (pI), net charge, and hydrophobicity were analyzed using the PepDraw (http://www.tulane.edu/~biochem/WW/PepDraw/, accessed on 16 December 2022) database. Potential biological activity was calculated using PeptideRanker (http://distilldeep.ucd.ie/PeptideRanker/, accessed on 16 December 2022).

### 2.8. Cell Culture Assays

#### 2.8.1. Cell Culture Growing Conditions

The HepG2 (HB-8065) human hepatocytes were obtained from the American Type Culture Collection (ATCC, Manassas, VA, USA) and cultured in Eagle’s Minimum Essential Medium (EMEM) supplemented with 10% fetal bovine serum (FBS), 1% penicillin-streptomycin at 37 °C, and 5% CO_2_. The cell viability was measured using the CellTiter 96 Aqueous One Solution Proliferation assay (Promega Corporation, Madison, WI, USA).

#### 2.8.2. Cell Model of Metabolic-Associated Fatty Liver Disease

Hepatocytes were cultured for 24 h seeded in flasks at a density of 5 × 10^5^ cells cm^−2^. Then, they were incubated in EMEM supplemented with 10% FBS in the absence (non-treated cells controls, NT) or presence of FFAs (500 μmol L^−1^ oleic:palmitic acid, 2:1) conjugated in BSA (1%), and the digested soybean varieties (V1, V3, V9, V17, and V18; 10–1000 μg protein mL^−1^) or simvastatin (0.10–10 μg mL^−1^; 0.24–24 μmol L^−1^). After 24 h treatment, supernatants were collected, centrifuged, and stored at −80 °C until being used as described earlier [29]. Once the half maximal inhibitory concentration (IC_50_) for cholesterol concentration increase was determined, cells were cultivated in the absence (non-treated cells controls, NT) or presence of FFAs (500 μmol L^−1^ oleic:palmitic acid, 2:1) and the digested soybean varieties (V1, V3, V9, V17, and V18 at IC_50_s 253, 160, 76, 64, and 146 µg protein mL^−1^, respectively) or simvastatin (IC_50_ = 160 ng mL^−1^). The treated cells were rinsed twice with ice-cold PBS, and then RIPA Lysis Buffer System (Santa Cruz Biotechnology, CA, USA) was added to lyse the cells. The cell suspension was sonicated and centrifuged at 10,000× *g* for 10 min at 4 °C to eliminate cell debris. Simultaneously, different aliquots were mixed with 4× Laemmli buffer, boiled for 5 min, and then either frozen or directly stored at −80 °C until further analysis. The protein concentration in cell lysates was quantified with the DC protein assay (BioRad).

#### 2.8.3. Assessment of Cellular HMGCR Activity

Intracellular HMGCR activity was measured in cell lysates of hepatocytes treated under the conditions described in Section 2.8.2. As previously explained (Section 2.6.), an HMGCR assay kit was utilized under the manufacturer’s recommended conditions. (Sigma-Aldrich). In summary, NADPH and HMG-CoA in a phosphate buffer pH 7.4 medium were placed into a UV-compatible 96-well plate. The analyses were initiated (time 0) by adding cell lysates (10 µL). A blank containing cell lysates buffer was included. The rates of NADPH consumed were monitored every 30 s for up to 20 min by reading the decrease in absorbance at 340 nm.

#### 2.8.4. Assessment of Cellular Cholesterol and Triglyceride Content

Intracellular hepatic total cholesterol, free, and esterified cholesterol, and triglyceride levels were determined in HepG2 cell lysates by enzymatic colorimetric kits (No. 10007640 and No. 10010303, respectively; Cayman Chemical, Ann Arbor, MI, USA). A blank containing cell lysates buffer was included in all assays. Cholesterol increase was defined as the relative increase in total intracellular cholesterol concentration upon FFA stimulation.

#### 2.8.5. In Silico Molecular Docking

Peptides produced during the simulated gastrointestinal digestion of the five selected varieties were analyzed as potential ligands for HMGCR and PCSK9 through molecular docking. The 3D crystal structures of HMGCR (2Q1L) and PCSK9 (7S5H) were obtained from the Protein Data Bank website (https://www.rcsb.org/pdb/home/home.do, accessed on 16 December 2022). Instant MarvinSketch was used to create peptide structures (ChemAxon Ltd., Boston, MA, USA). AutoDock Tools was used to combine non-polar hydrogen atoms, add Gasteiger partial charges, and specify the root of each structure’s rotatable bonds. Furthermore, AutoDock Tools was used to determine the docking space dimensions (HMGCR: 26 × 22 × 20; PCSK9: 32 × 24 × 28), center point, and flexible torsions. The docking space center was selected based on the location of the co-crystallized inhibitor (HMGCR: *x*, 34.419; *y*, −18.502; *z*, 7.371; PCSK9: *x*, 36.887; *y*, 88.637; *z*, 168.806). One hundred runs were performed for each ligand, and the conformation with the best binding mode was used to calculate the ligand binding energy (*ΔG*) using PRODIGY [30]. Each peptide’s ligand–protein interactions were examined using Discovery Studio 2017 R2 Client (Dassault Systèmes Biovia Corp, San Diego, CA, USA). The interaction constants ($K_{i}$) were determined using Equation (3).

(3) $$K_{i}{\;(\mathrm{mol}\;L}^{-1}{)=e}^{\left(1000\;\times \;\Delta {G\;R}^{-1}T^{-1}\right)}$$

where *ΔG* is the peptide binding energy, *R* is the gas constant (cal K^−1^ mol^−1^), and *T* is the absolute temperature (K).

#### 2.8.6. Cholesterol Metabolism-Related Protein Expression by Western Blot

Similar quantities of cell lysate protein (15 μg) were separated by electrophoresis using 4–20% gradient SDS-PAGE gels. Separated proteins were transferred onto PVDF membranes (ref. 88518, Thermo Scientific, Rockford, IL, USA), which were then blocked with 5% (*w/v*) nonfat dry milk in Tris-buffered saline + 0.1% Tween 20 for 1 h at room temperature. Membranes were incubated overnight at 4 °C with primary mouse antibodies for human p-AMPK^T172^ (2535, Cell Signaling, Danvers, MA; epitope in T172), AMPK (sc-74461; epitope in amino acids 251–550), SREBP-2 (sc-271616, epitope in amino acids 812–975), HMGCR (sc-271595, epitope in amino acids 589–888), SIRT1 (sc-74504, epitope in amino acids 448–747), p-ACC^S78/S80^ (sc-271965 epitope in amino acids S78 and S80), ACC (sc-137104, epitope in amino acids 1–76), SREBP-1c (sc-17755, epitope in amino acids 41–200), FASN (sc-48357, epitope in amino acids 2205–2504), LDLR (sc-18823, epitope in amino acids 13–47), and PCSK9 (sc-515082, epitope in amino acids 175–334) purchased in Santa Cruz Biotechnology, unless otherwise stated. All the membranes were then washed and probed with secondary sheep anti-mouse antibodies (1:5000, 1 h, RT; GE Healthcare, Buckinghamshire, UK). SuperSignal™ West Femto maximum sensitivity chemiluminescent (ECL) substrate (Invitrogen) was used to reveal the protein bands, and then pictures were obtained on an ImageQuant 800 System (GE Healthcare, Buckinghamshire, UK). Protein loading controls (GAPDH, sc-47724) were used to calculate the relative expression of each protein.

#### 2.8.7. Very Low-Density Lipoprotein (VLDL) Release through Apolipoprotein B (ApoB) Measurement

The ApoB levels in the collected media were determined with a human ApoB ELISA development kit (MABTECH, Inc., Stockholm, Sweden; catalog no.: 3715-1H-6) in 96-well ELISA plates (Thermo Scientific, Rockford, IL, USA; catalog no.: 07-200-640) with 3,3′,5,5′ tetramethylbenzidine substrate (Thermo Fisher Scientific; catalog no.: 4041). The ApoB concentration was calculated with the ApoB standard provided by the manufacturer in parallel on the same plate.

#### 2.8.8. Lipid Accumulation

Intracellular lipid accumulation was investigated in HepG2 cells seeded in 96-well plates, kept in complete growth medium for 24 h, and then treated with FFAs (500 μmol L^−1^ oleic:palmitic acid, 2:1) and digested soybeans at their IC_50_ for cholesterol concentration increase (V1, V3, V9, V17, and V18 at 253, 160, 76, 64, and 146 µg protein mL^−1^, respectively) or simvastatin (160 ng mL^−1^) for another 24 h. After treatments, cells were washed twice with PBS, fixed with formalin (4%), rinsed again with PBS, and incubated with Nile Red (1 μg mL^−1^) and NucBlue™ reagent (Hoechst 33342, Invitrogen) for 20 min. Residual staining was eliminated with PBS, and the fluorescent signal was measured under the following conditions (excitation/emission): 530⁄590 nm for the lipid content and 360⁄460 nm for the nuclei content. Lipid accumulation levels were normalized with the number of cells according to the nuclei signal. Then, representative images were collected using a Cytation5 Cell Imaging Multi-Mode Reader (BioTek, Winooski, VT, USA).

#### 2.8.9. Angiopoietin-like Protein 3 (ANGPTL3) Measurement

The levels of extracellular ANGPTL3 protein in treated cells media samples were measured using a human ANGPTL3 ELISA Kit (RayBiotech, Norcross, GA, USA) according to the manufacturer’s instructions. The absorbance was measured at 450 nm.

#### 2.8.10. Lipoprotein Lipase (LPL) Activity Measurement

The extracellular proteins in the culture medium of the treated cells were concentrated with a centrifugal concentrator MWCO 10 kDa (Sigma-Aldrich, St. Louis, MO, USA). LPL activity was measured using a fluorometric assay according to the manufacturer’s instructions kit (Cell Biolabs, San Diego, CA, USA). Briefly, the LPL (15.625 mU mL^−1^), the concentrated cell supernatants (50 μg of protein), and fluorescent substrates were incubated at 37 °C for 90 min on a 96-well plate (black bottom). The fluorescence intensities were measured at 485/528 nm, excitation/emission, respectively.

#### 2.8.11. LDL Uptake in Hepatocytes

To evaluate LDL uptake, HepG2 cells were seeded in 96-well plates, kept in complete growth medium for 24 h and then treated with FFAs (500 μmol L^−1^ oleic:palmitic acid, 2:1) and digested soybean varieties at their IC_50_ for cholesterol concentration increase (V1, V3, V9, V17, and V18 at 253, 160, 76, 64, and 146 µg protein mL^−1^, respectively) and simvastatin (160 ng mL^−1^) for another 24 h. At the end of the treatment, the culture medium was replaced with 10 μg mL^−1^ well LDL-Dil (1,1′-dioctadecyl-3,3,′,3′-tetramethylindocarbocyanine perchlorate) solution (Kalen Biomedical, Germantown, MD, USA). The cells were additionally incubated for 2 h at 37  °C. Finally, the culture medium was aspirated and replaced with PBS. Cells were stained with Hoechst 33342 (Invitrogen) for 20 min and washed with PBS. The degree of LDL uptake was measured using a fluorescent plate reader (excitation and emission wavelengths 540 and 570 nm, respectively). The nuclei fluorescence intensity (360/460 nm, excitation/emission) was used to normalize the values. Representative images were collected using a Cytation5 Cell Imaging Multi-Mode Reader (BioTek).

### 2.9. LDL Oxidation Assay

#### 2.9.1. In Plate Oxidation of LDL

LDL oxidation was evaluated in plate, using an in vitro biochemical cell-free assay. Human LDL (50 µg protein mL^−1^; Kalen Biomedical) were incubated in a medium containing phosphate buffer (10 mmol L^−1^, pH 7.4) and different concentrations of digested soybean (0.3–1000 µg protein mL^−1^) for 5 min at 37 °C. Afterward, the oxidation was initiated by the addition of CuSO_4_ (final concentration 10 µmol L^−1^) in the reaction medium. Early and end products from lipid peroxidation chain reactions were monitored by the formation of conjugated dienes (CD) or malondialdehyde (MDA) and 4-hydroxynonenal (HNE), respectively.

#### 2.9.2. Assessment of Lipid Early Peroxidation

CD formation was monitored spectrophotometrically by changes in the absorbance at 234 nm. Absorbance was measured each 2 min (0–240 min). Kinetic curves were fitted to nonlinear regression curves using the Gompertz growth equation [31]. The maximum oxidation rate, given by the peak of the first derivative, i.e., change in absorbance at 234 nm as a function of time. The percentage of CD formation inhibition was calculated by integrating the area under the kinetic curve for each soybean concentration (∫S_AUC_) and then normalizing with the area of the non-treated control (∫C_AUC_) following Equation (4).

(4) $$\mathrm{CD}\;\mathrm{formation}\;\mathrm{inhibition}\;(\%)=\left(1\;-\frac{\int S_{\mathrm{AUC}}\;}{\int C_{\mathrm{AUC}}}\right)\times \;100$$

Since values obtained from tested digests were relative to that of the control from the same experimental run, variations caused by LDL susceptibility difference, instrument sensitivity, or reagents can be minimized. Finally, the dose required to cause a 50% inhibition of LDL oxidation (IC_50_) was calculated.

#### 2.9.3. Assessment of Lipid Late Peroxidation

MDA and HNE were quantified in the reaction media after 240 min incubation using a commercial kit (KB03002, Bioquochem, Oviedo, Spain). Results were expressed as µmol equivalents of 1,1,3,3-tetramethoxypropane L^−1^.

### 2.10. Statistical Analysis

Data were expressed as means of at least three independent replicates. For comparisons between soybean varieties, data were analyzed by one-way analysis of variance (ANOVA) and post hoc Tukey’s test. Differences were considered significant at *p* < 0.05. Univariate and bivariate analysis (Pearson correlations; see Appendix A, Table A1) of the results was performed with SPSS 26.0. Principal component analysis (PCA) and hierarchical cluster analysis were used to classify digested soybean varieties according to their protein profile and cholesterol-regulating and LDL oxidation-preventive properties. Multivariate analysis (PCA and hierarchical clustering) was computed using XLSTAT2021. Graphs were depicted using GraphPad Prism 9.0 (GraphPad Software, Inc., San Diego, CA, USA).

## 3. Results and Discussion

### 3.1. Soybean Varieties Exhibited a Differential Protein Hydrolysis during Gastrointestinal Digestion

**Figure 1:**
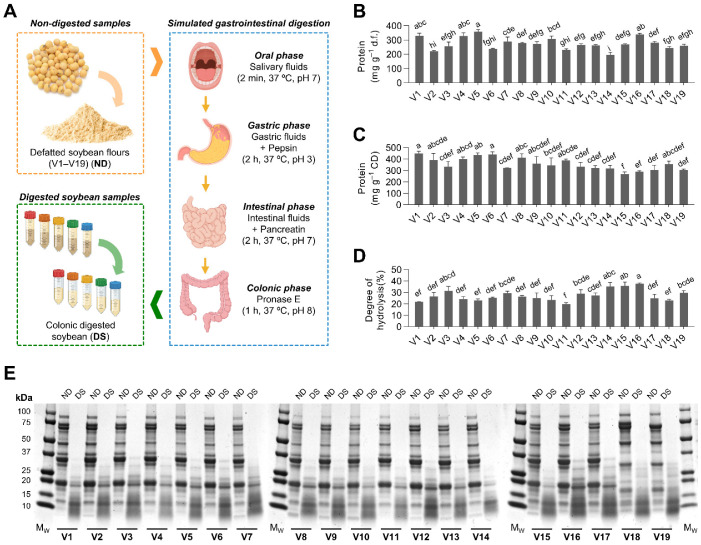
Illustrative diagram of the simulated digestion process followed to hydrolyze the different soybean varieties flours (V1 to V19) (**A**). Protein concentration (mg g^−1^) in defatted soybean flours (d.f.) (**B**) and colonic digested soybeans (DS) (**C**). Degree of hydrolysis of the digested soybean flours (**D**) and representative SDS-PAGE electrophoresis gels (**E**) of the proteins from non-digested (ND) defatted soybean flours and their colonic digested soybeans (DS). Results are reported as mean ± SD (*n* = 3). Bars with different letters significantly differ according to ANOVA and Tukey’s multiple range test (*p* < 0.05).

The defatted flour of nineteen different soybean varieties was subjected to gastrointestinal digestion, including oral, gastric, duodenal, and colonic steps (Figure 1A). The protein concentration in non-digested soybean defatted flour ranged from 196 to 357 mg g^−1^ soybean flour (Figure 1B). Notwithstanding the discovery of numerous grain protein-controlling quantitative trait loci linked to soybean protein accumulation, both concentration and profile are highly influenced by environmental factors [32,33]. Soybean peptides are released during gastrointestinal digestion due to the action of acids and digestive enzymes from the stomach, small intestine, and pancreas. Furthermore, lactic acid bacteria in the gastrointestinal system produce bioactive peptides via their protease activity [34,35]. Protein concentration in digested soybean varieties (257–450 mg g^−1^ digested soybean) was not associated with protein concentration in the defatted flour (Figure 1C). Other soybean components found in different concentrations in the nineteen soybean varieties (polysaccharides, phenolic compounds, saponins) may be distinctively released during gastrointestinal digestion and, therefore, influence protein concentration in the digested soybeans [36]. Furthermore, interactions between minor soybean components (isoflavones) and soybean proteins (mainly glycinin and β-conglycinin) may alter protein physicochemical properties and digestibility [37].

The degree of hydrolysis varied (*p* < 0.05) among samples, from 20% in V11 to 37% in V16 (Figure 1D). Protein digestibility in soybean flours can be influenced by several factors, such as the food matrix structure (particle size, cell wall integrity, or the involvement of other components), soybean cooking processes (soaking, boiling, or fermentation), and the composition and structure of soybean proteins [36,38]. Soybeans, traditionally recognized as a nutrient-dense source of dietary protein, are composed of several storage proteins with diverse characteristics. Among these proteins, glycinin and β-conglycinin have been proposed to account for several of the biological effects of soybeans [39]. SDS-PAGE electrophoresis gels demonstrated that the nineteen soybean varieties were composed of different proportions of proteins (Figure 1E; Appendix A, Figure A1). Glycinin proportion varied from 22% in V18 to 60% in V11, whereas β-conglycinin ranged 21–52%, being higher in V18 and V19, and lower in V9, V10, and V17. Yang et al. [40] found similar glycinin (14–49%) and β-conglycinin (16–42%) proportions when analyzing 93 different soybean varieties. Battisti et al. [41] proved that β-conglycinins were the most variable protein in amount in soybean milk, whereas glycinins were less variable. Since other proteins, such as protease inhibitors, are less abundant, their variability is rather limited [42]. Additionally, we observed that until the colonic phase, gastrointestinal digestion yielded hydrolysates with, in general, no residual undigested proteins over 25 kDa. Digested soybean varieties V1–V17 showed a band between 15 and 20 kDa, which did not appear in V18–V19. This fact could indicate that this polypeptide may have resulted from glycinin hydrolysis, considering the low concentration of glycinin in soybean varieties V18–V19. A similar protein hydrolysis pattern was observed by Nguyen et al. [43], who reported a progressive hydrolysis of β-conglycinin over gastrointestinal digestion, an accumulation of small polypeptides (<20 kDa), and the presence of a strong band, putatively associated with undigested glycinin.

### 3.2. Digested Soybean Could Be Classified according to Their Composition and Capacity to Inhibit HMGCR

**Figure 2:**
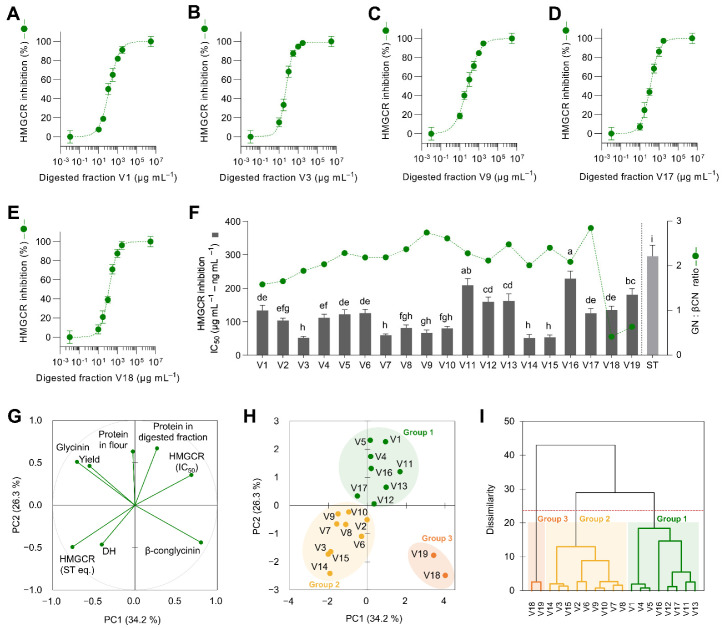
Dose–response curves of the 3-hydroxy-3-methylglutaryl-CoA reductase (HMGCR) inhibitory effect of different soybean varieties digested under gastrointestinal conditions (V1: (**A**); V3: (**B**); V9: (**C**); V17: (**D**); and V18: (**E**)) and the half-inhibitory concentration (IC_50_, µg protein mL^−1^; ng simvastatin mL^−1^) of digested soybean in relation to their glycinin:β-conglycinin ratio (**F**). Principal component (PC) analysis (loadings in (**G**) and PC scores in (**H**)), including the composition of digested soybean varieties and their HMGCR inhibitory properties. Dendrogram of the hierarchical cluster analysis classifying samples (**I**). Results are reported as mean ± SD (*n* = 3). Bars with different letters significantly differ according to ANOVA and Tukey’s multiple range test (*p* < 0.05). The dotted line in panel (**F**) separates soybean varieties from simvastatin due to the difference in their IC_50_ units.

The nineteen soybean flour varieties digested under simulated gastrointestinal conditions were tested for their inhibition of the activity of HMGCR. Figure 2A–E shows five representative dose–response curves for HMGCR inhibition. Both IC_50_ for the nineteen soybean varieties and glycinin:β-conglycinin ratios are presented in Figure 2F. The glycinin:β-conglycinin ratio varied from 0.42 (V18) to 2.85 (V17). Yang et al. [40] observed that in over 93 soybean varieties, the glycinin:β-conglycinin ratio fluctuated from 0.46 to 2.73. The glycinin:β-conglycinin ratio might be of great practical significance for variety selection to fit their specific food and health applications. As hypothesized, soybean activity is not only associated with different protein concentrations but also with the composition of those proteins and the peptides that are embedded in them and released during gastrointestinal digestion. Nonetheless, there was no linear association between the glycinin:β-conglycinin ratio and the HMGCR inhibitory activity of digested soybeans. In plate, HMGCR activity inhibition may respond to the concentration of multiple proteins and peptides. IC_50_ varied from 59 to 229 µg protein mL^−1^. The inhibitory properties of digested soybean varieties were 199–774-fold lower than those of simvastatin (IC_50_ = 296 ng mL^−1^). Previous reports also described that peptic/tryptic soybean hydrolysates might inhibit HMGCR [16]. Other legume hydrolysates and pure peptides have also demonstrated HMGCR activity inhibition [44,45].

Multivariate analysis, PCA, and hierarchical cluster analysis classified the nineteen soybean varieties according to their chemical composition and HMGCR inhibitory properties (Figure 2G–I). PC1 (comprising 34.2% of the variability) was mainly influenced by the concentration of glycinin and β-conglycinin and the HMGCR inhibition (Figure 2G). By contrast, PC2 (26.3% of the variability) was mainly influenced by the protein concentration in the digested soybeans. PCA loadings demonstrated that glycinin and β-conglycinin concentrations were not associated with lower or higher HMGCR activity inhibition determined in plate. Both glycinin and β-conglycinin could contribute to HMGCR activity inhibition [13,15]. PC scores (Figure 2H) and the dendrogram (Figure 2I) classified samples into three different groups. Group 1 was characterized by higher protein concentrations and HMGCR IC_50_ (lower inhibition). Group 2 was characterized by a higher degree of hydrolysis and higher enzyme inhibition. Both groups exhibited similar levels of glycinin and β-conglycinin. On the contrary, group 3 was characterized by the highest concentration of β-conglycinin. Accordingly, five different representative varieties were selected for the following experiments (V1 and V17 from group 1; V3 and V9 from group 2; and V18 from group 3). V1 and V17, having similar HMGCR inhibitory properties (IC_50_ = 133 and 125 µg protein mL^−1^, respectively), differed in their glycinin:β-conglycinin ratio (1.6 and 2.8, respectively). V3 and V9 exhibited higher HMGCR inhibition (IC_50_ = 53 and 67 µg protein mL^−1^, respectively) but varied on their glycinin:β-conglycinin ratio (1.9 and 2.7, respectively). V18 showed a potential to inhibit HMGCR (IC_50_ = 135 µg protein mL^−1^) similar to V1 and V17 but was characterized by the lowest glycinin:β-conglycinin ratio (0.4).

### 3.3. Selected Soybean Varieties Yielded Different Peptide Profiles during Gastrointestinal Digestion

**Figure 3:**
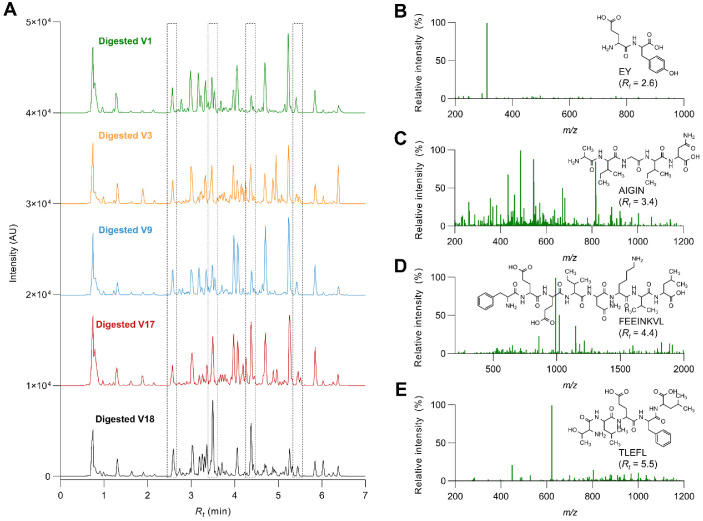
Representative chromatograms of the selected soybean varieties (**A**) and mass spectra of the main peaks (framed ones) identified in the soybean varieties digested under gastrointestinal conditions (**B**–**E**).

The digests from the selected five varieties were further characterized by LC-MS/MS, and de novo peptide sequencing was used to identify the peptides found in each digested variety. TIC chromatograms show the peptide profiles in the selected digested soybeans (Figure 3A). Representative mass spectra of four different peptides are represented in Figure 3B–E.

Thirteen different peptides were tentatively identified (seventeen when considering their L/I and flipped variants). Peptides found in the different varieties contained between two and eight amino residues (Table 1). Most of the identified peptides came from soybean storage proteins (glycinin, β-conglycinin, and basic 7S globulin) but also from protease inhibitors (Kunitz trypsin inhibitor) and allergens (P34 and profilin). As observed, most of them were produced from the hydrolysis of any of the subunits of β-conglycinin. As observed in the SDA-PAGE electrophoresis, glycinin might be less digestible than β-conglycinin; therefore, fewer peptides would be released from that protein. The molecular weight of the peptides ranged from 243 to 991 Da. The isoelectric point varied from 3.1 to 11.1, the net charge from −1 to +2, and the hydrophobicity from 4.45 to 14.27 kcal mol^−1^. Peptides were also ranked according to their potential bioactivity. NKLGK exhibited the lowest bioactivity (15.0%) and GPA the highest (75.5%).

Although soybean peptides have been detected in plasma, quantitative assays are not available. Sato et al. [46] reviewed maximum plasmatic concentration (*C_max_*) after food/hydrolysate/peptide consumption, concluding that the peptide’s structure affects its intestinal absorption and metabolic fate. Zhang et al. [47] investigated the bioavailability of a soybean peptide in rats. The peptide’s *C_max_* was 130 µg mL^−1^. Considering that 20–200-fold *C_max_* doses produced accurate results when measuring compounds’ effects in vitro [48], in our research, we tested soybean digests between 10 and 1000 μg protein mL^−1^.

### 3.4. Digested Selected Soybean Varieties Protect Liver Cells from the Free Fatty Acid Challenge

**Figure 4:**
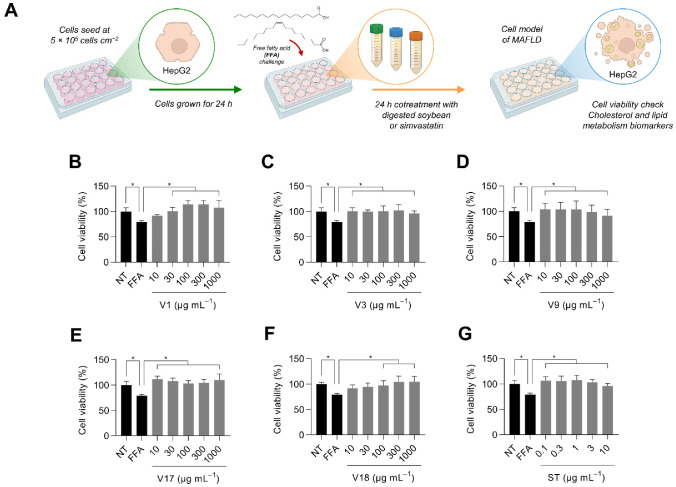
Illustrative diagram of the experimental design followed for evaluating the metabolic effects of digested soybean in liver cells under metabolic-associated fatty liver disease (MAFLD) (**A**). Basal cell viability of selected digested soybean varieties (**B**–**F**) and simvastatin (**G**). Results are reported as mean ± SD (*n* = 3). Bars that significantly (*p* < 0.05) differ according to ANOVA and Dunnet’s multiple range test are marked with a superscript asterisk (*). NT: non-treated cells; FFA: cells treated with free fatty acids (500 µmol L^−1^ oleic: palmitic acid, 2:1).

**Figure A2:**
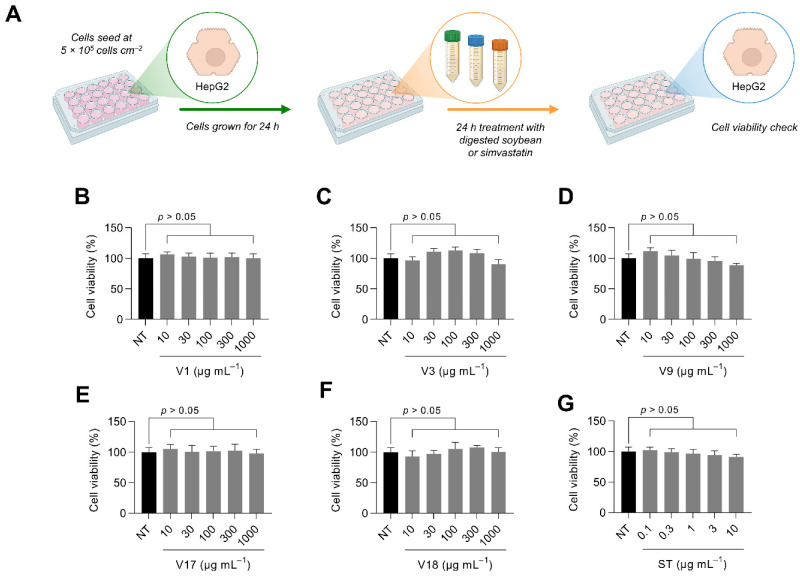
Illustrative diagram of the experimental design followed for evaluating cell viability under basal conditions (**A**). Basal cell viability of selected digested soybean varieties (**B**–**F**) and simvastatin (**G**). Results are reported as mean ± SD (*n* = 3).

To study the hypocholesterolemic effects of the selected five digested soybean varieties further, we evaluated the effects of different concentrations of the digested fractions (V1, V3, V9, V17, V18; 10–1000 µg protein mL^−1^) and the drug control (simvastatin, 0.1–10 µg mL^−1^) in human HepG2 liver cells (Appendix A, Figure A2A). None of the treatments modified the cell viability under the 24 h administration of these soybean digests (Figure A2B–G). Then, these concentrations were further used for the in vitro model of MAFLD. Similarly, cells were treated with digested soybean but in the presence of free fatty acids (500 µmol L^−1^ FFA, oleic: palmitic acid, 2:1) (Figure 4A). Although the FFA challenge significantly reduced cell viability (*p* < 0.05), the digested soybean fraction counteracted FFA’s detrimental effects, at least at 100 µg mL^−1^. Comparably, simvastatin (0.1–10 µg mL^−1^) prevented (*p* < 0.05) the loss in cell viability (Figure 4B–G).

### 3.5. Peptides from Selected Soybean Varieties Reduced Cholesterol Synthesis by Inhibiting HMGCR

**Figure 5:**
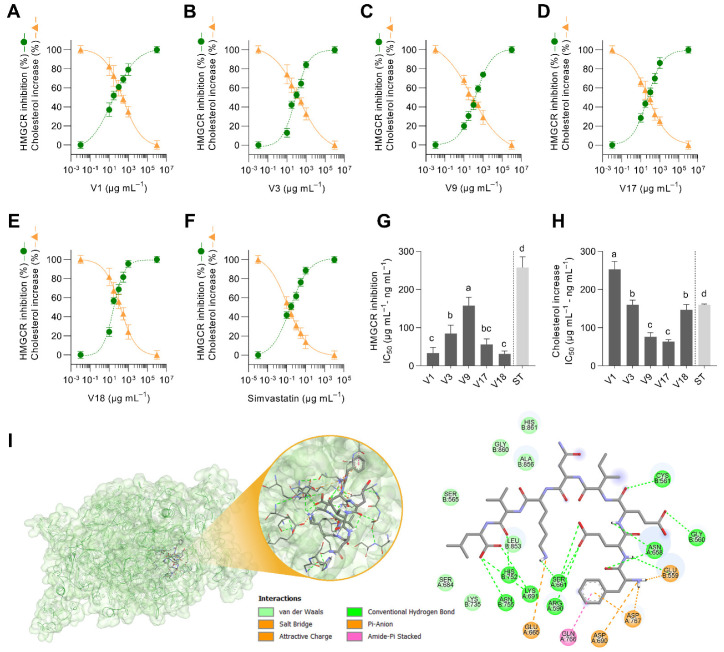
Dose–response curves of the effect of selected soybean digested varieties (**A**–**E**) and simvastatin (ST) (**F**) on the 3-hydroxy-3-methylglutaryl-CoA reductase (HMGCR) activity and cholesterol concentration increase in free fatty acid-stimulated HepG2 liver cells (500 µmol L^−1^ oleic: palmitic acid, 2:1) and their half-inhibitory concentration (IC_50_, µg protein mL^−1^; ng simvastatin mL^−1^) on HMGCR activity (**G**) and cholesterol concentration increase (**H**). Representative HMGCR-peptide (FEEINKVL) in silico interaction in the active site (**I**). Results are reported as mean ± SD (*n* = 3). Bars with different letters significantly differ according to ANOVA and Tukey’s multiple range test (*p* < 0.05). The dotted line in panels (**G**,**H**) separates soybean varieties from simvastatin due to the difference in their IC_50_ units.

The HMGCR activity was evaluated in FFA-treated cells to confirm the hypocholesterolemic effects observed in the cell-free model. In addition, the concentration of total intracellular cholesterol was measured (Figure 5). The selected soybean varieties (V1, V3, V9, V17, and V18) and simvastatin-reduced cholesterol concentration increased and inhibited HMGCR in a dose–response manner (Figure 5A–F). HMGCR inhibitory properties were again variety-dependent. As observed in Figure 5G,H, the IC_50_ values for the five selected soybean varieties differed significantly (*p* < 0.05). Varieties V1, V17, and V18 inhibited better HMGCR (lower IC_50_), followed by V17 and V3.

The IC_50_ values for HMGCR inhibition significantly correlated with the concentration of glycinin (*r* = 0.525, *p* < 0.05) and negatively with the β-conglycinin levels (*r* = −0.594, *p* < 0.05). Then, the presence of β-conglycinin seemed to benefit the inhibition of HMGCR. The cholesterol concentration increase was inversely correlated to the inhibition of HMGCR (*r* = −0.806, *p* < 0.05). The higher hypocholesterolemic and HMGCR inhibitory effects of β-conglycinin in comparison to glycinin were previously demonstrated in vivo [49,50]. Figure 5I depicts the interaction of FEEINKVL, the peptide with the strongest binding energy (−10.5 kcal mol ^−1^), with the active site of HMGCR. The active site of HMGCR, where HMG-CoA is reduced, is located in a cis-loop where the main catalytic residues are Lys^691^, Glu^559^, Asp^767^, and His^866^ [51]. The interaction was stabilized via multiple hydrogen bonds (Gly^560^, Cys^561^, Arg^590^, Asn^655^, Ser^661^, Lys^691^, His^752^, Asn^755^), attractive charges and salt bridges (Glu^559^, Glu^665^, Asp^690^, Asp^767^), π-type interactions (Gln^766^, Asp^767^), and van del Waals interactions. Similarly, simvastatin interacted with HMGCR’s active site thought hydrogen bonds (Arg^590^, Lys^691^, Asn^755^) and alkyl interactions (Leu^562^, His^752^, Ala^856^, Leu^853^, Leu^857^). Both molecules shared some hydrogen bonds, indicating that their interactions might be similarly effective. All the other peptides released from selected soybean varieties under gastrointestinal conditions exhibited binding energies between −6.9 and −9.2 kcal mol ^−1^ and interaction constants between 0.02 and 8.75 µmol L^−1^ (Table 2). Further research is needed, however, to understand the molecular pathway underlying the observed effects. Then, to deepen understanding of the mechanism of action of the selected soybean varieties, HepG2 liver cells were further treated at their IC_50_ for cholesterol concentration increase.

### 3.6. Digested Soybean Varieties Inhibited Cholesterol Esterification and ApoB Secretion by Modulating AMPK Phosphorylation

**Figure 6:**
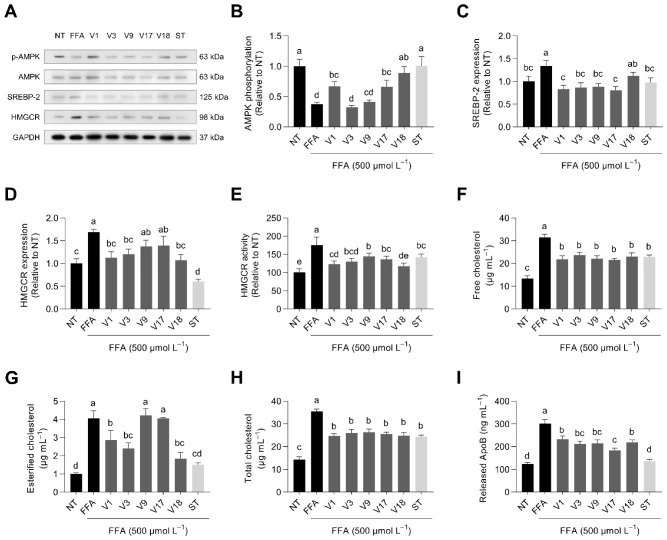
Effect of selected soybean digests (V1, V3, V9, V17, and V18 at 253, 160, 76, 64, and 146 µg protein mL^−1^, respectively) and simvastatin (ST, 160 ng mL^−1^) on the relative protein expression/phosphorylation (**A**) of AMP-activated protein kinase (AMPK) as p-AMPK/AMPK ratio (**B**), sterol regulatory element-binding protein 2 (SREBP-2) (**C**), and 3-hydroxy-3-methylglutaryl-CoA reductase (HMGCR) (**D**), HMGCR relative activity (**E**), and the intracellular concentration of free (**F**), esterified (**G**), and total (**H**) cholesterol and the release of apolipoprotein B (ApoB) (**I**) in free fatty-acid-stimulated HepG2 liver cells (500 µmol L^−1^ oleic: palmitic acid, 2:1). Results are reported as mean ± SD (*n* = 3). Bars with different letters significantly differ according to ANOVA and Tukey’s multiple range test (*p* < 0.05).

Cholesterol synthesis is tightly regulated by the sterol regulatory element-binding protein 2 (SREBP-2) as a transcriptional regulator and the rate-limiting enzyme of cholesterol biosynthesis, HMGCR [52]. In turn, AMP-activated protein kinase (AMPK), considered a master switch regulator of lipid metabolism involved in the control of multiple cellular processes, inhibits SREBP-2 and HMGCR, therefore modulating cholesterol homeostasis [53]. Those proteins were regulated in FFA-stimulated hepatocytes co-treated with digested soybean varieties (Figure 6A). AMPK phosphorylation was reduced (63%) by the FFA challenge (Figure 6B). This effect was repressed by soybean digests V1, V17, V18, and simvastatin (15–100%). 

SREBP-2 precursor expression increase (1.3-fold) was also counterbalanced by soybean varieties (63–100%) (Figure 6C). SREBP-2 expression negatively correlated with the glycinin content (*r* = −0.942, *p* < 0.001). Other reports have proven that the FFA-triggered SREBP-2 precursor expression would be associated with an increased expression of the SREBP-2 mature and active form, tightly linked to the subsequent expression of HMGCR and the enhancement in cholesterol synthesis [54,55]. The augmented expression (1.7-fold) (Figure 6D) and activity (1.8-fold) (Figure 6E) of HMGCR were also regulated by soybean varieties (33–83% and 28–65%, respectively) after triggering hepatocytes with the FFA cocktail. HMGCR expression negatively correlated with the glycinin:β-conglycinin ratio (*r* = 0.947, *p* < 0.001). However, the HMGCR expression and activity determined in cells did not correlate with the in-plate HMGCR activity inhibition, presumably due to the changes caused by absorption and metabolism in the cell culture model.

Upon the FFA treatment, liver cells viewed their intracellular concentration of free cholesterol increase 2.4-fold (Figure 6F). Selected digested soybean varieties inhibited (43–55%) free cholesterol concentration increases. Similarly, the concentration of esterified cholesterol increased 4.1-fold (*p* < 0.05), but some digested soybean varieties (V1, V3, and V18) reduced it (39–73%) as well as simvastatin (84%) (Figure 6G). Esterified cholesterol increases negatively correlated with the concentration of β-conglycinin (*r* = −0.869, *p* < 0.001). Similarly, esterified cholesterol correlated with the glycinin:β-conglycinin ratio (*r* = 0.929, *p* < 0.001). Adams et al. [56] reported significant decreases in aortic cholesteryl ester levels after a 16-week administration of β-conglycinin, compared with soybean protein/isolate, glycinin, or low β-conglycinin soybean protein, in an in vivo model of atherosclerosis. Intracellular total cholesterol increased 2.5-fold (Figure 6H) due to the presence of FFA. Cholesterol synthesis was inhibited by all digested soybean varieties and simvastatin (43–52%). Total cholesterol increase was significantly associated with the glycinin:β-conglycinin ratio (*r* = 0.711, *p* < 0.001). The enzyme acyl-CoA cholesterol acyltransferase (ACAT) catalyzes intracellular cholesterol esterification, which is a crucial mechanism for preventing excessive cellular amounts of free cholesterol, which can be harmful to cells [57]. After esterification, cholesterol may be accumulated in lipid droplets or transferred to ApoB-containing triglyceride-rich lipoprotein particles, such as VLDL [58]. In our study, the release of ApoB into the cell medium was increased (2.4-fold) by the FFA stimulation. Selected soybean varieties reduced ApoB secretion by about 39–67% and simvastatin by 93% (Figure 6I). Lovati et al. [59] reported positive effects of soybean protein and peptides on reducing ApoB secretion in HepG2 cells. In addition, Pipe et al. [60] demonstrated that soybean protein intake reduced serum apolipoprotein B. Similarly, Ma et al. [61] observed a reduction in ApoB plasmatic levels in hyperlipidemic women after consuming β-conglycinin for 12 weeks.

### 3.7. Selected Digested Soybeans Reduced de Novo Lipogenesis via AMPK-SIRT1 Activation

**Figure 7:**
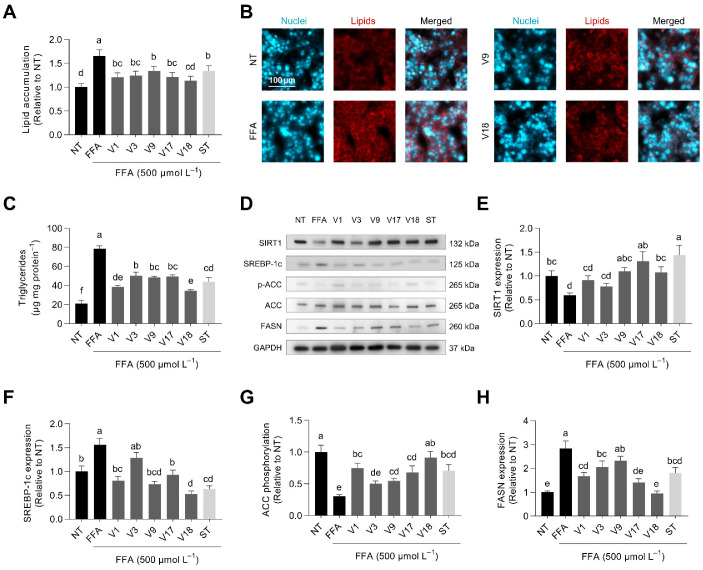
Effect of selected soybean digests (V1, V3, V9, V17, and V18 at 253, 160, 76, 64, and 146 µg protein mL^−1^, respectively) and simvastatin (ST, 160 ng mL^−1^) on the intracellular lipid accumulation (**A**,**B**), triglyceride concentration (**C**), the relative protein expression/phosphorylation (**D**) of sirtuin 1 (SIRT1) (**E**), sterol regulatory element-binding protein 1c (SREBP-1c) (**F**), acetyl-CoA carboxylase (ACC) (**G**), and fatty acid synthase (FASN) (**H**) in free fatty-acid-stimulated HepG2 liver cells (500 µmol L^−1^ oleic: palmitic acid, 2:1). Results are reported as mean ± SD (*n* = 3). Bars with different letters significantly differ according to ANOVA and Tukey’s multiple range test (*p* < 0.05).

MAFLD is characterized by excessive fat deposition in the form of triglycerides in the liver (steatosis) [62]. As observed in Figure 7A,B, FFA elicited a 1.7-fold increase in the intracellular content of lipids. Digested soybean prevented lipid accumulation by 49–79% (*p* < 0.05). FFA-treated HepG2 hepatocytes exhibited 3.7-fold higher intracellular triglycerides than non-treated cells (Figure 7C). Soybean varieties (51–70%) and statin (60%) significantly (*p* < 0.05) prevented the accumulation of triglycerides. Lipid homeostasis is controlled through multiple nutrient sensors, such as sirtuin 1 (SIRT1), AMPK, acetyl-CoA carboxylase (ACC), sterol regulatory element-binding protein 1c (SREBP-1c), and fatty acid synthase (FASN) [63]. We observed modulation of those proteins’ expression in hepatocytes challenged with FFA (Figure 7D). SIRT1 protein expression was reduced (60%) in response to the FFA treatment (Figure 7E). Soybean varieties (V9, V17, and V18) prevented SIRT1 protein decrease (45–100%), overstimulating it with V17 and statin treatments. SIRT1, together with AMPK, mediates the biological response of cells to nutrient availability [64]. AMPK inhibits de novo lipogenesis by regulating ACC, which catalyzes the rate-limiting step in fatty acid synthesis by converting acetyl-CoA to malonyl-CoA. AMPK activation also reduces the expression of SREBP-1c and its downstream gene, FASN [65]. FFA augmented SREBP-1c precursor expression 1.6-fold, but soybean varieties (V1, V9, V17, and V18) fully inhibited it (Figure 7F). Increased FFA-derived SREBP-1c precursor expression in hepatocytes is linked to a rise in the expression of its mature form, which, after its cleavage in the Golgi apparatus, translocates into the nucleus where it activates target gene expression (i.e., ACC and FASN) [66,67]. Here, we observed a 30% reduced ACC phosphorylation after FFA stimulation. Selected soybean varieties prevented FFA effects by 35–88% (Figure 7G). ACC phosphorylation was negatively associated with the glycinin concentration (*r* = −0.831, *p* < 0.001). Comparably, FFA-triggered and exacerbated (2.8-fold) FASN expression was reduced (43–100%, *p* < 0.05) by digested soybean varieties (V1, V3, V17, V18) (Figure 7H). FASN expression negatively correlated with the concentration of β-conglycinin (*r* = −0.732, *p* < 0.01). 

Previous research also indicated that β-conglycinin in soybeans might be associated with reduced FASN expression in adipocytes [24]. Interestingly, simvastatin reduced the intracellular triglyceride concentration. Previous reports demonstrated that low simvastatin concentrations might regulate HepG2 triglyceride content by activating AMPK and SIRT1 signaling pathways [68]. Indirectly, the accumulation of HMG-CoA could inhibit FASN, therefore contributing to a reduced lipid accumulation [69]. Accordingly, soybean-digests-triggered activation of AMPK and SIRT1 might exert a pivotal role in lipogenesis regulation, governing the activation of SREBP-1c and, thence, the expression of ACC and FASN—the main enzymes regulating hepatic de novo lipogenesis under the MAFLD-mimicking microenvironment [64].

### 3.8. Digested Soybean Reduced ANGPTL3 Release, Therefore, Preserving LPL Activity

**Figure 8:**
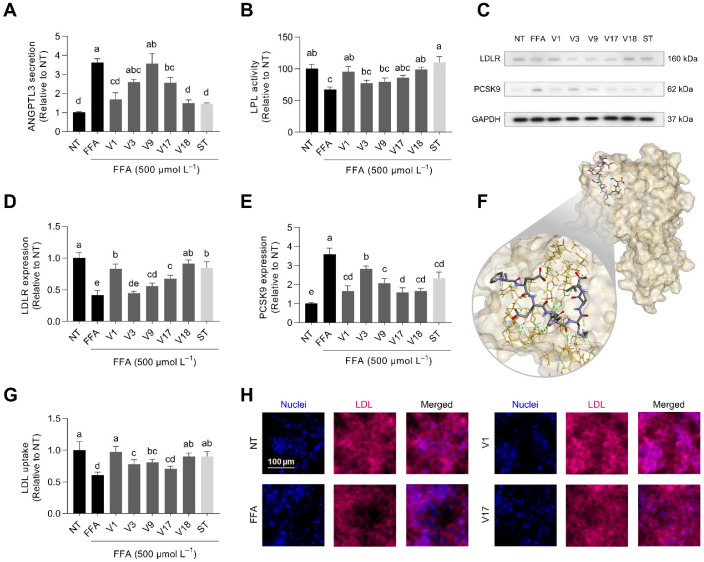
Effect of selected soybean digests (V1, V3, V9, V17, and V18 at 253, 160, 76, 64, and 146 µg protein mL^−1^, respectively) and simvastatin (ST, 160 ng mL^−1^) on the secretion of angiopoietin-like 3 (ANGPTL3) (**A**), the activity of lipoprotein lipase (LPL) (**B**), the relative protein expression (**C**) of the LDL receptor (LDLR) (**D**) and proprotein convertase subtilisin/kexin type 9 (PCSK9) (**E**) in free fatty-acid-stimulated HepG2 liver cells (500 µmol L^−1^ oleic: palmitic acid, 2:1). Representation of the PCSK9-peptide (FEEINKVL) in silico interaction in the LDLR binding site (**F**). LDL uptake in hepatocytes co-treated with FFA and selected soybean varieties digested under gastrointestinal conditions (**G**,**H**). Results are reported as mean ± SD (*n* = 3). Bars with different letters significantly differ according to ANOVA and Tukey’s multiple range test (*p* < 0.05).

ANGPTL3 is a hepatokine primarily produced in the liver and then released into the bloodstream in response to agonists of the liver X receptor (LXR) [70]. Since ANGPTL3, together with ANGPTL8, inhibits LPL, which is the main enzyme involved in the hydrolysis of triglyceride-rich lipoproteins, augmented ANGPTL3 secretion may decrease the lipolysis of VLDL and LDL and result in a slower turnover of lipoprotein remnants and increased plasmatic ApoB levels [71]. We observed increased ANGPTL3 levels (3.6-fold) after the FFA challenge (Figure 8A). Digested V1, V17, and V18 significantly reduced ANGPTL3 secretion (41–81%, *p* < 0.05). ANGPTL3 release correlated with the glycinin:β-conglycinin ratio (*r* = 0.827, *p* < 0.001). Concurrently, the activity of LPL, reduced (33%) by the presence of FFA, was protected by selected soybean varieties V1 and V18 (86 and 97%, respectively) (Figure 8B). The increases in ANGPTL3 negatively correlated with the loss of LPL activity (*r* = −0.871, *p* < 0.01), however, other extracellular factors, including ANGPTL8, secreted from FFA-stimulated HepG2 cells might also contribute to LPL inhibition [72]. LPL activity negatively correlated with the glycinin proportion in soybean (*r* = −0.766, *p* < 0.01). There is scarce information on the effects of dietary bioactive compounds on the regulation of ANGPTL3. Some phenolic compounds, flavones, and xanthones have demonstrated their ability to reduce the expression of ANGPTL3 and increase LPL activity [73,74]. Interventional studies have also proven that pecan, cotton, or olive oil consumption might also modulate plasmatic ANGPTL3 [75,76]. Finding bioactive compounds that may regulate plasmatic ANGPTL3 levels and activity is guiding the latest research relative to the prevention and treatment of hyperlipidemia and atherosclerosis [77].

### 3.9. Digested Soybean Stimulated LDL Uptake via Regulation of LDLR and PCSK9

LDL clearance is mediated by LDLR. Increased LDLR expression improves LDL hepatic absorption and decreases plasma LDL. Conversely, PCSK9 functions as a chaperone, guiding the LDLR to internal degradation and preventing its recycling to the cell surface. [78]. Digested soybean varieties counteracted FFA’s adverse effects on LDLR and PCSK9 expression (Figure 8C). The expression of LDLR was reduced by 68% after FFA treatment (Figure 8D). Soybean digests prevented LDLR reduction by 16–81%. The expression of LDLR negatively correlated with the proportion of glycinin in selected soybean varieties (*r* = −0.739, *p* < 0.01). Similarly, LDLR expression inversely correlated with HMGCR activity (*r* = −0.704, *p* < 0.05) and ANGPTL-3 (*r* = −0.796, *p* < 0.01). Elevated HMGCR activity and ANGPTL-3 release are associated with diminished LDLR expression and LDL uptake in the liver. Since those proteins are overexpressed under MAFLD conditions, regulating them using food compounds may represent a nutritional strategy to prevent atherosclerotic cardiovascular diseases derived from high cholesterol and LDL levels [8,79]. Conversely, the expression of PCSK9 was augmented 3.2-fold (Figure 8E). Digested soybean varieties prevented this increase (17–90%). PCSK9 participates in cholesterol homeostasis by initiating the intracellular degradation of the LDLR after binding to it and consequently decreasing blood LDL clearance [80]. The expression of LDLR negatively correlated with the expression of PCSK9 (*r* = −0.829, *p* < 0.001). Previous reports demonstrated that peptides from major soybean proteins could stimulate LDLR and inhibit PCSK9 [18,19]. Moreover, peptides released from the selected soybean varieties under gastrointestinal digestion conditions could interact with the LDLR binding site (Table 2). Binding energies varied from −6.7 to −8.8 kcal mol^−1^, whereas the constant for PCSK9–peptide interaction ranged 0.35–12.27 µmol L^−1^. Figure 8F illustrates the best binding pose of FEEINKVL with PCSK9. The epidermal growth factor precursor homology domain A of the LDLR binds to the surface PCSK9 between aminoacidic residues 367–381. Key interaction sites between the LDLR and PCSK9 subtilisin-like catalytic domain involve residues Arg^194^ and Asp^238^, Asp^374^, and Phe^379^ [81]. The interaction of FEEINKVL with the PCSK9 surface was stabilized by several hydrogen bonds (Lys^222^, Ser^225^, Cys^255^, Asp^374^, Phe^379^, Ser^381^, Gln^382^, Ser^383^), carbon–hydrogen bonds (Ser^372^), π-π staked and van del Waals interactions. Comparably, the synthetic inhibitor (a cyclic peptide) interacted though hydrogen bonds (Phe^379^, Ser^381^), carbon–hydrogen bonds (Asp^238^, Thr^377^, Cys^378^, Val^380^), halogen interactions (Val^380^), alkyl (Ile^369^), and π-type interactions. Then, the similarities between the interacting residues profiles indicate the potential of this FEEINKVL to behave as a PCSK9 inhibitor. Consistently, FFA elicited a reduction (39%) in LDL hepatic uptake (Figure 8G,H). Digested soybean inhibited hepatic reduced LDL absorption by 25–92%. Soybean peptic/tryptic hydrolysates have also denoted LDL-uptake-stimulating properties in HepG2 cells [16]. Therefore, digested soybean varieties might promote plasmatic LDL reductions by triggering hepatic LDL uptake via inhibiting PCSK9.

### 3.10. Soybean Varieties Digested under Gastrointestinal Conditions Inhibited LDL Oxidation

**Figure 9:**
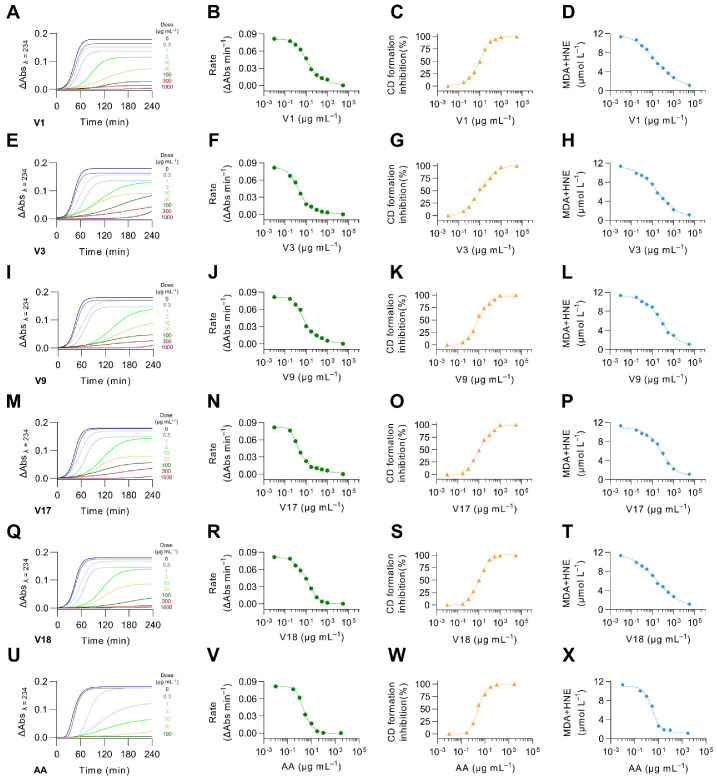
Effect of selected soybean digests (V1, V3, V9, V17, and V18 at 0.3–1000 µg protein mL^−1^) and ascorbic acid (AA, 0.3–1000 µg mL^−1^) on LDL oxidation markers: kinetic changes in absorbance at 234 nm (0–240 min), rate constant of the reaction propagation (K, ΔAbs min^−1^), and the formation of conjugated dienes (CD, % inhibition relative to control), malondialdehyde and 4-hydroxynonenal (MDA+HNE, µmol L^−1^). Panels (**A**–**D**,**E**–**H**,**I**–**L**,**M**–**P**,**Q**–**T**,**U**–**X**) correspond to LDL oxidation markers of soybean varieties V1, V3, V9, V17, V18, and ascorbic acid, respectively. Results are reported as mean ± SD (*n* = 3). Bars with different letters significantly differ according to ANOVA and Tukey’s multiple range test (*p* < 0.05).

Oxidized LDL is the most prominent risk factor in atherosclerotic cardiovascular diseases [82]. Soybean-derived peptides have demonstrated in vitro and in vivo antioxidant properties [83]. Therefore, we investigated the effects of selected digested soybean varieties on preventing LDL oxidation (Figure 9). We evaluated the kinetics of LDL oxidation throughout 240 min at eight different concentrations of digested soybean varieties. Increasing concentrations of the digested soybean varieties and ascorbic acid (used as a control in this assay) reduced the increase in the medium absorbance (λ = 234). Furthermore, the LDL oxidation rate was reduced by all digested soybean varieties in a dose-dependent manner. The selected varieties of digested soybean effectively inhibited the formation of CD (early oxidation products) and MDA+HNE (late oxidation products) (Table 3). The IC_50_ for the formation of CD ranged from 8.1–11.3 µg protein mL^−1^, whereas the IC_50_ for MDA+HNE production varied from 19.70 to 70.2 µg protein mL^−1^. Ascorbic acid, a potent natural antioxidant, exhibited a higher inhibition of LDL oxidation. IC_50_ for CD and MDA production was 2.6 and 0.9 µg mL^−1^, respectively. The glycinin:β-conglycinin ratio correlated with CD formation (*r* = 0.675, *p* < 0.01) and MDA+HNE synthesis (*r* = 0.856, *p* < 0.001). Previous studies demonstrated that the 3-month intake of high β-conglycinin soybean milk reduced the levels of oxidized LDL in overweight men to a greater extent than in regular soybean milk [84].

### 3.11. Soybean Varieties with Lower Glycinin:β-Conglycinin Ratio Were Associated with a Higher Potential to Regulate Hepatic Lipid Metabolism and LDL Homeostasis

**Figure 10:**
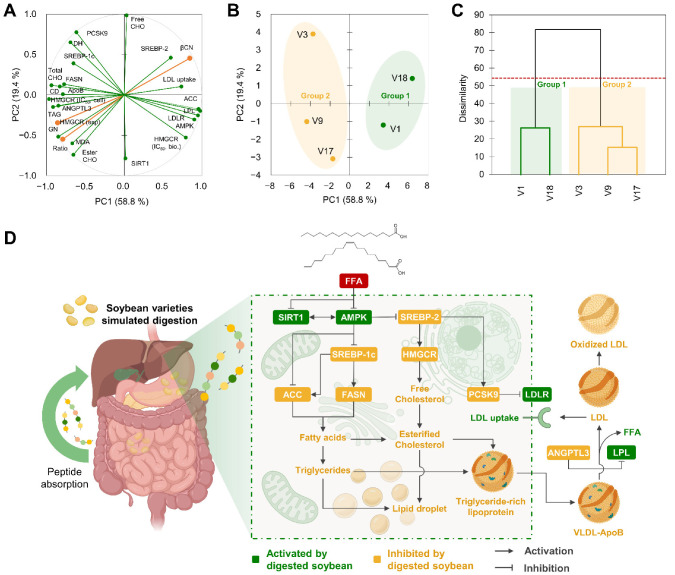
Principal component (PC) analysis (loadings in (**A**) and PC scores in (**B**)), including the composition of soybean varieties, digests, HMGCR inhibitory properties, and hypocholesterolemic effects in free fatty-acid-stimulated liver cells. Dendrogram of the hierarchical cluster analysis classifying samples (**C**). Illustrative diagram summarizing the main signaling pathways modulated by the different soybean varieties digested under gastrointestinal conditions on hepatic lipid and cholesterol-LDL metabolism and oxidation (**D**).

Multivariate analysis investigated the association among all the parameters analyzed (Figure 10A–C). PCA loadings showed a similar pattern as observed for the starting nineteen varieties (Figure 10A). PC1, accounting for 58.8% of the variability, mainly included the influence of glycinin and β-conglycinin concentration, the inhibition of HMGCR, the concentration of total cholesterol and intracellular triglycerides, and the expression of LDRL, AMPK, ACC, as the most significant factors. Other factors associated with this first component included: ANGPTL3 expression and LPL activity, SREBP-1c and FASN expression, and CD and MDA, as markers of LDL oxidation. PC2 (19.4% of the variability) was mainly influenced by the concentration of free and esterified cholesterol and the expression of SIRT1 and PCSK9. In general, PC1 and PC2 explained 78.2% of the variability. Another two components were found, mainly associated with the protein concentration in non-digested and digested soybeans. Hence, cell culture measures evidenced the association of the glycinin:β-conglycinin ratio in digested soybean varieties and the modulation of hepatic cholesterol hemostasis and the regulation of LDL oxidation and clearance. This way, PC scores (Figure 10B) and the hierarchical cluster analysis (Figure 10C) categorized samples into two groups. Group 1 included V1 and V18, with the highest β-conglycinin proportions (among the five selected varieties) and the highest inhibition of cholesterol synthesis, HMGCR activity, lipogenesis, and LDL oxidation and recycling. Group 2 sorted V3, V9, and V17, with higher glycinin proportion and degree of hydrolysis and higher HMGCR regulatory properties determined in plate.

Figure 10D summarizes the mechanism underlying the effects of digested soybean varieties on triglyceride and cholesterol homeostasis under MAFLD conditions. Upon the FFA challenge, digested soybean counteracted the lost AMPK/SIRT1 expression, thereby activating SREBP-1c and SREBP-2. Selected soybean varieties reduced the exacerbated HMGCR activity triggered by SREBP-2 overexpression. As a result, cholesterol synthesis was reduced. Concurrently, digested soybeans hindered the elicited SREBP-1c, leading to enhanced ACC and FASN expression. Afterward, selected soybean varieties blocked the synthesis of fatty acids, their esterification into triglycerides and cholesterol, and the further formation of lipid droplets or triglyceride-rich lipoproteins (VLDL). Then, soybean digests may prevent steatosis by reducing de novo lipogenesis and attenuating the progress of MAFLD. Concomitantly, the release of ApoB was reduced, indicating a diminished secretion of triglyceride and cholesterol-rich VLDL particles. The lessened release of ANGPTL3 favored LPL activity, favoring the recycling of LDL through its hepatic uptake. Reduced PCSK9 expression prompted LDLR expression, therefore stimulating LDL absorption into the liver. Finally, digested soybean varieties functioned as radical scavengers preventing the oxidation of LDL particles. Taken together, these results indicate that the intake of selected soybean varieties might regulate cholesterol and LDL homeostasis and, consequently, foster the prevention of atherosclerotic cardiovascular diseases.

Despite the potential in vitro biological activity of the peptides released from the selected soybean varieties under simulated gastrointestinal conditions, their limited bioavailability constrains their effectiveness [85]. Peptides and other bioactive compounds potentially released from soybean during digestion and possibly contributing to the investigated effects, such as phenolic compounds and saponins, may reach the liver and bloodstream in a different chemical form [86]. Several studies have indicated that only peptides with specific structures can be transferred to the blood flow at the micromolar level upon ingestion. Some food-derived peptides might be metabolized into active compounds in the body. Thus, it is crucial to identify food-derived peptides and their metabolites in the target organs [46]. To date, clinical studies targeting MAFLD with soybean have included a small number of participants and a short intervention, which constrained the statistical significance of these investigations that did not show significant effects on the lipid metabolism of MAFLD patients [11]. We do not discard that future investigations targeting MAFLD by the daily soybean intake will validate the results observed in vitro in the present study. Therefore, in vivo and human studies will be required to prove the triglyceride-, cholesterol-, and LDL-regulating effects observed in vitro and determine the absorption and metabolism of soybean proteins and peptides.

## 4. Conclusions

This study found that the soybean variety affects the protein composition and peptide release under simulated gastrointestinal conditions. In turn, protein composition influenced the hypocholesterolemic properties of soybean varieties digested under gastrointestinal conditions. We present, for the first time, the hypolipidemic potential of the digested soybeans in a cell model of MAFLD. Digested soybean varieties reduced cholesterol synthesis in human liver cells by inhibiting HMGCR activity, decreased lipogenesis by reducing ACC and FASN expression, reduced VLDL release, promoted LDL clearance by reducing ANGPTL3 and PCSK9 expression and by prompting LDLR expression, and finally inhibited LDL oxidation. These effects were associated with the differential proportion of glycinin and β-conglycinin in soybean proteins, i.e., their glycinin:β-conglycinin ratio. Remarkably, the greater the proportion of β-conglycinin in soybean varieties digested under gastrointestinal conditions, the more the resulting peptides reduced HMGCR expression, the concentration of esterified cholesterol and triglycerides, the release of ANGPTL3, and the production of MDA during LDL oxidation. Then, soybean digested under gastrointestinal conditions may modulate cholesterol and lipid metabolism in hepatocytes based on the glycinin:β-conglycinin ratio. The outcomes of this study, based on results from an in vitro model of MAFLD using liver cells, provide new insight into the potential beneficial effects of soybean on cardiovascular health. Soybean intake may help to regulate cholesterol homeostasis in the liver and LDL oxidation, improving the potential for cardiovascular health. Soybean ingredients made from soybeans with greater proportions of β-conglycinin may be useful to inspire foods and meals containing synergistic components that together can improve the potential for healthful outcomes.

## Figures and Tables

**Table 1:** Peptides identified in selected soybean varieties digests and their physicochemical and potential biological properties **^‡^**.

R_t_ (min)	Peptide Sequence	Parental Protein	Mass (Da)	pI	Net Charge	Hydrophobicity (kcal mol^−1^)	Bioactivity (%)	Found in
1.0	GPA	Kunitz trypsin inhibitor	243.12	5.60	0	9.69	75.5	V1, V3, V9, V17, V18
1.2	LR	β-conglycinin α’	287.20	11.11	+1	8.46	57.0	V1, V3, V9, V17, V18
		β-conglycinin α 1/2						
		β-conglycinin β 1/2						
		Glycinin G4						
	RL	β-conglycinin α’	287.20	10.73	+1	8.46	62.6	
		β-conglycinin α 1/2						
		β-conglycinin β 1/2						
		P34						
	IR	β-conglycinin α’	287.20	11.12	+1	8.59	33.8	
		β-conglycinin α 1/2						
		β-conglycinin β 1/2						
		Glycinin G4						
		Basic 7S globulin						
		P34						
		Profilin-1						
	RI	β-conglycinin α’	287.20	10.73	+1	8.59	33.2	
		β-conglycinin α 1/2						
		β-conglycinin β 1/2						
		Glycinin G4						
		Basic 7S globulin						
1.6	AHAI	P34	410.23	7.95	0	10.11	18.8	V1, V3, V9, V17, V18
1.9	FR	Glycinin G4	321.18	10.90	+1	8.00	98.6	V1, V3, V9, V17
2.6	EY ^†^	Basic 7S globulin	310.12	3.14	−1	10.82	6.68	V1, V3, V9, V17, V18
3.4	FE	β-conglycinin α’	294.12	3.14	−1	9.82	59.9	V1, V3, V9, V17, V18
		β-conglycinin α 1/2						
		Glycinin G4						
		P34						
	EF	β-conglycinin α’	294.12	3.09	−1	9.82	59.0	
		β-conglycinin α 1/2						
		β-conglycinin β 1/2						
3.5	AIGIN ^†^	β-conglycinin α 1/2	486.28	5.42	0	8.16	29.9	V1, V3, V9, V17, V18
3.7	RALS	β-conglycinin α 1/2	445.26	10.73	+1	9.42	18.7	V1, V3, V9, V17, V18
4.4	FEEINKVL ^†^	β-conglycinin α’ β-conglycinin α 1/2 β-conglycinin β 1/2	990.54	4.09	−1	14.27	17.1	V1, V3, V9, V17, V18
4.7	NKLGK	β-conglycinin α’ β-conglycinin α 1	558.35	10.56	+2	14.25	15.0	V1, V9, V17, V18
5.3	GVAW	Glycinin G1 Glycinin G2	431.22	5.70	0	7.0	63.6	V1, V3, V9, V17
5.4	AIVIL	β-conglycinin α 1/2 β-conglycinin β 1/2	527.37	5.59	0	4.45	23.5	V1, V3, V9, V17, V18
5.5	TLEFL ^†^	Glycinin G1	621.34	3.20	−1	7.57	32.4	V3, V17

^†^ Representative peptides whose mass spectra are shown in Figure 3. ^‡^ Physicochemical properties were computed in https://pepdraw.com/, accessed on 16 December 2022. Potential biological activity was ranked in http://distilldeep.ucd.ie/PeptideRanker/, accessed on 16 December 2022.

**Table 2:** Binding energy (*ΔG*) and constant of interaction (*K_i_*) between 3-hydroxy-3-methylglutaryl-CoA reductase (HMGCR), proprotein convertase subtilisin/kexin type 9 (PCSK9), and the peptides identified in the selected soybean varieties digested under gastrointestinal conditions.

Peptide	HMGCR		PCSK9	
	*ΔG* (kcal mol ^−1^)	*K_i_* (µmol L^−1^)	*ΔG* (kcal mol ^−1^)	*K_i_* (µmol L^−1^)
GPA	−7.2	5.28	−6.9	8.75
LR	−7.0	7.39	−6.9	8.75
RL	−7.0	7.39	−6.7	12.27
IR	−7.0	7.39	−6.9	8.75
RI	−6.9	8.75	−6.7	12.27
AHAI	−7.7	2.27	−7.4	3.76
FR	−7.4	3.76	−7.0	7.39
EY	−7.9	1.62	−7.5	3.18
FE	−7.9	1.62	−7.3	4.46
EF	−8.1	1.16	−7.6	2.69
AIGIN	−8.0	1.37	−7.7	2.27
RALS	−7.5	3.18	−7.1	6.25
FEEINKVL	−10.5	0.02	−8.8	0.35
NKLGK	−8.2	0.98	−7.4	3.76
GVAW	−8.5	0.59	−7.7	2.27
AIVIL	−8.8	0.35	−7.6	2.69
TLEFL	−9.2	0.18	−8.0	1.37
Simvastatin	−9.7	0.08	−9.0	0.25
PCSK9 inhibitor ^†^	—	—	−9.4	0.13

^†^ The PCSK9 inhibitor was not docked against HMGCR, indicated as “—”.

**Table 3:** Inhibitory activity (IC_50_, µg mL^−1^) of selected soybean digests (V1, V3, V9, V17, and V18, 0.3–1000 µg protein mL^−1^) and ascorbic acid (AA, 0.3–1000 µg mL^−1^) on LDL oxidation markers: formation of conjugated dienes (CD), malondialdehyde and 4-hydroxynonenal (MDA+HNE).

Soybean Variety	CD	MDA+HNE
V1	8.1 ± 0.6 ^c^	19.7 ± 5.0 ^c^
V3	10.9 ± 0.8 ^ab^	30.0 ± 6.8 ^b^
V9	10.3 ± 1.0 ^ab^	66.1 ± 9.6 ^a^
V17	11.3 ± 0.7 ^a^	70.2 ± 9.9 ^a^
V18	9.0 ± 0.6 ^bc^	22.9 ± 4.9 ^bc^
AA	2.6 ± 0.5 ^d^	0.9 ± 0.1 ^d^

Results are reported as mean ± SD (*n* = 3). Rows with different letters significantly differ according to ANOVA and Tukey’s multiple range test (*p* < 0.05).

## Data Availability

Data are contained within the article.

## Appendix A

**Table A1:** Pearson correlations between the concentration of glycinin and β-conglycinin, and the glycinin: β-conglycinin ratio and the evaluated biochemical and cell culture markers of cholesterol metabolism and LDL oxidation.

Variables	Glycinin Concentration		β-Conglycinin Concentration		Glycinin:β-Conglycinin Ratio	
	*r*	*p*-Value	*r*	*p*-Value	*r*	*p*-Value
HMGCR (IC_50_, Biochemical assay)	−0.497	0.060	0.454	0.089	−0.389	0.152
HMGCR (IC_50_, Cell culture)	0.525	0.044	−0.594	0.020	0.627	0.012
AMPK phosphorylation	−0.743	0.002	0.696	0.004	−0.618	0.014
SREBP-2 expression	**−0.942**	**<0.001**	**0.911**	**<0.001**	**−0.821**	**<0.001**
HMGCR expression	0.789	<0.001	**−0.848**	**<0.001**	**0.947**	**<0.001**
HMGCR activity	−0.794	<0.001	0.739	0.002	−0.690	0.004
Free cholesterol concentration	−0.416	0.123	0.516	0.049	−0.542	0.037
Esterified cholesterol concentration	0.778	0.001	**−0.869**	**<0.001**	**0.929**	**<0.001**
Total cholesterol concentration	0.644	0.010	−0.654	0.008	0.711	0.003
VLDL-Apolipoprotein B release	0.388	0.153	−0.426	0.113	0.543	0.036
Lipid accumulation	0.734	0.002	−0.789	<0.001	0.764	0.001
Triglycerides concentration	**0.880**	**<0.001**	**−0.847**	**<0.001**	**0.867**	**<0.001**
SIRT1 expression	0.078	0.783	−0.183	0.514	0.370	0.175
ACC phosphorylation	**−0.831**	**0.000**	0.791	<0.001	−0.734	0.002
SREBP-1c expression	0.644	0.010	−0.507	0.054	0.413	0.126
FASN expression	0.726	0.002	−0.732	0.002	0.643	0.010
ANGPTL3 release	0.724	0.002	−0.777	0.001	**0.827**	**<0.001**
LPL activity	−0.766	0.001	0.731	0.002	−0.729	0.002
LDLR expression	−0.739	0.002	0.683	0.005	−0.657	0.008
PCSK9 expression	0.308	0.264	−0.208	0.458	0.132	0.640
LDL uptake	−0.591	0.020	0.564	0.029	−0.688	0.005
CD formation (IC_50_)	0.605	0.017	−0.567	0.027	0.675	0.006
MDA formation (IC_50_)	0.636	0.011	−0.717	0.003	**0.856**	**<0.001**

Pearson correlation coefficients (*r*) higher than ± 0.8 are highlighted in bold.
